# Precision Nursing: The Personalized Future of Care and Revolution in Clinical Practice

**DOI:** 10.1590/0034-7167-2025-0423

**Published:** 2026-03-30

**Authors:** Luís Carlos Lopes-Júnior

**Affiliations:** I Department of Noncommunicable Diseases and Mental Health, Pan American Health Organization - World Health Organization. Washington, D.C., United States of America

**Keywords:** Precision Nursing, Precision Medicine, Biomarkers, Symptom Science, Personalized Care., Enfermería de Precisión, Medicina de Precisión, Biomarcadores, Ciencia de los Síntomas, Cuidado Personalizado.

## Abstract

**Objectives:**

to discuss the foundations, applications, challenges, and perspectives of precision nursing in contemporary practice, with an emphasis on the incorporation of genomic technologies, biomarkers, artificial intelligence and symptom science.

**Methods:**

a theoretical-reflective study based on recent scientific evidence.

**Results:**

nurses occupy a strategic position to lead the translation of precision medicine knowledge into clinical practice, especially in symptom management and personalized decision-making. Ongoing studies on inflammatory biomarkers, gastrointestinal disorders, symptomatic distress, and the use of digital devices in self-care stand out. Ethical, structural, and training challenges persist, such as curricular reformulation, professional training, and equity in access to innovations.

**Final Considerations:**

precision nursing can transform care by enabling more effective, safe, and person-centered interventions. It is essential to strengthen data science, foster translational nursing research, and promote its gradual integration into care practice.

## RESUMEN

**Objetivos:**

analizar los fundamentos, las aplicaciones, los desafíos y las perspectivas de la enfermería de precisión en la práctica contemporánea, con énfasis en la incorporación de tecnologías genómicas, biomarcadores, inteligencia artificial y ciencia de los síntomas.

**Métodos:**

estudio teórico-reflexivo basado en evidencia científica reciente.

**Resultados:**

el personal de enfermería ocupa una posición estratégica para liderar la aplicación del conocimiento de la medicina de precisión a la práctica clínica, especialmente en el manejo de síntomas y la toma de decisiones personalizada. Cabe destacar los estudios en curso sobre biomarcadores inflamatorios, trastornos gastrointestinales, distrés sintomático y el uso de dispositivos digitales en el autocuidado. Persisten desafíos éticos, estructurales y formativos, como la reformulación curricular, la formación profesional y la equidad en el acceso a las innovaciones.

**Consideraciones Finales:**

la enfermería de precisión puede transformar la atención al permitir intervenciones más efectivas, seguras y centradas en la persona. Es fundamental fortalecer la ciencia de datos, fomentar la investigación traslacional en enfermería y promover su integración gradual en la práctica asistencial.

## INTRODUCTION

Researchers around the world seek to translate advances from the laboratory bench into clinical practice and healthcare systems^(1)^. Building on structural genomics, new techniques have enabled the development of functional genomics, which integrates with “omics” sciences, including proteomics, epigenomics, transcriptomics, pharmacogenomics, and metabolomics. These approaches investigate variations in the human genome functioning throughout development and in different environmental contexts, enhancing knowledge about the molecular mechanisms of disease^(1)^.

The growing understanding of molecular biology has driven the transition to precision medicine (PM), a new paradigm based on personalizing treatment according to individual or population genetic/genomic characteristics^(2)^. PM recognizes that different groups have distinct genomic profiles and proposes more effective, safe and cost-effective therapeutic interventions^(1,2)^. This approach aims to deliver the right medication, in the right dose, at the right time, overcoming the limitations of the traditional one-size-fits-all treatment model, which can be ineffective and lead to adverse effects^(2)^.

PM advancement also influences nursing practice, highlighting precision nursing (PN) as an emerging field that reinforces nurses’ role in the coordination, implementation, and translation of personalized, person-centered care^(3)^. Nurses, because of their proximity to patients and specialized clinical knowledge, are in a strategic position to lead this translation^(3)^. However, significant challenges still need to be overcome to consolidate PN in clinical practice, including professional training, integration of genomic technologies into daily care, and equity in access to these innovations^(3,4)^.

Given this scenario, it is essential that nursing embraces PM and PN advances, expanding its scope to provide increasingly personalized, predictive, preventive, and participatory care. Incorporating these approaches into clinical practice can transform healthcare, promote more effective interventions and improve patient outcomes.

## OBJECTIVES

To discuss the foundations, applications, challenges, and perspectives of PN in contemporary practice, with an emphasis on the incorporation of genomic technologies, biomarkers, artificial intelligence, and symptom science.

## METHODS

This is a theoretical-reflective study, developed based on the author’s critical analysis, grounded in recent and relevant scientific evidence on PN. The text was constructed through the following stages: (1) bibliographic survey in MEDLINE/PubMed, Embase, Web of Science, and Scopus, using controlled descriptors and keywords related to “precision nursing”, “precision medicine”, “biomarkers”, “symptom science”, “personalized care”, “personalized nursing care”, and “omics sciences”; (2) selection of articles, technical documents, and guidelines published in the last ten years, prioritizing systematic reviews, high-quality primary studies, position papers from scientific societies, and reports from international organizations; (3) analytical reading and identification of central thematic categories; and (4) critical integration of evidence with the author’s professional and academic experience, aiming to discuss the foundations, applications, challenges, and perspectives of the topic. This approach allowed us to articulate the available scientific literature with original reflections, favoring a broader and prospective understanding of PN in the contemporary context.

## RESULTS

### Scientific Advances in Precision Nursing: Applications of Research in Clinical Practice

Nurses around the world have been expanding their work in identifying biomarkers associated with different conditions, such as chronic stress in children and adolescents, frailty in older adults, vascular access maintenance, pressure injury prevention, and cognitive impairment^(5,6)^. Other nursing professionals have explored artificial intelligence to predict the risk of falls and pressure injuries as well as investigating biomarkers in non-pharmacological interventions to optimize clinical outcomes^(7-9)^. The focus on prevention, management and symptom relief is one of the main contributions of nursing science to precision health, aimed at reducing symptoms and improving quality of life^(9,10)^. Incorporating this concept into nursing research is essential to improving the development and application of interventions across the entire continuum of care^(9,10)^.

A study explored the observed racial differences in relapse rates of children with acute lymphoblastic leukemia (ALL) to highlight the need to incorporate multiple factors into PM when studying clinical outcomes of the disease^(11)^. Although the significant role of genetics in ALL is emphasized, other factors, such as social determinants of health, also influence care accuracy^(11)^. This study highlights the need for collaborative and transdisciplinary research to investigate the complex interactions between multiple factors that contribute to PM.

Based on the National Institute of Nursing Research (NINR) Symptom Science Model^(12)^, NINR-funded P20/30 Centers play a key role in incorporating symptom science into the different precision health domains. These initiatives encompass omics approaches, behavioral science, and self-care, as well as considering the social determinants of health for a more comprehensive understanding of clinical conditions^(9,12)^.

Several nurse scientists have developed research that uses precision health to improve understanding of symptom burden, predict and prevent diseases, as well as optimize individualized treatments and patients’ quality of life^(9-13)^. One example is a study conducted at NINR that investigates the mechanisms involved in symptomatic distress in digestive disorders, with an emphasis on the biobehavioral relationships between inflammation and symptoms^(14)^. This research seeks to identify the biological and behavioral processes that influence the manifestation of symptoms, highlighting the brain-gut-microbiota axis’s role^(14)^. The findings indicate that chronic gastrointestinal symptoms may be associated with subclinical inflammatory mechanisms^(14)^.

To enable the full development of precision health, it is essential to strengthen the data science infrastructure at both the institutional and national levels. Traditionally, clinical data are obtained through methods such as standardized questionnaires, healthcare service registries, electronic medical records, omics reports, imaging tests, and physiological monitoring. However, emerging technologies such as mobile health (mHealth) devices, non-invasive sensors like actigraphy, and passive monitoring systems have been incorporated to provide a more detailed analysis of individuals’ biological, behavioral, and environmental characteristics^(9,11)^. A practical example of this advancement can be seen in diabetes research, where the integration of different data sources allows for a more comprehensive phenotype characterization. This process takes into account factors such as lifestyle, environment, genetic profile, and biomarkers. This information is essential for personalizing interventions, making symptom management and self-care more effective for individuals more likely to respond positively to therapeutic strategies^(15)^.

### Incorporating Precision Nursing into Clinical Practice: Strategies for Nurses’ Daily Life

PN is not an approach that is distant from nurses’ daily practice; on the contrary, it can be integrated into care in a simple and progressive manner, enhancing personalized care and optimizing health outcomes^(3,6,16)^. For this to happen, nurses must adopt strategies that facilitate PN incorporation into different care settings. Chart 1 illustrates the main strategies for implementing of PN in clinical practice.

**Chart 1:** Strategies for implementing precision nursing, Washington, D.C., United States of America, 2025

Strategies for implementing PN in nursing clinical practicescrição	
1. Qualified Collection and Recording of Patient Data	PN’s foundation is personalized care, and this is only possible with detailed and accurate patient data. Nurses can improve clinical information collection by considering: 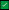 Detailed health history, including genetic predisposition, family history, and environmental factors; 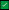 Lifestyle and daily habits, such as nutrition, physical activity, sleep patterns, and stress levels; 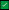 Social and psychological factors, which can influence response to treatment and therapeutic adherence; 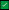 Use of technologies, such as electronic medical records, non-invasive devices, and monitoring applications, to record and track data in a structured manner.
2. Use of Biomarker-Based Screening Tools and Artificial Intelligence	PN allows nurses to use biomarkers and technological resources to anticipate risks, predict complications, and personalize interventions. Practical examples include: 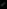 Monitoring of inflammatory biomarkers (e.g., C-reactive protein, neutrophil-lymphocyte ratio, platelet-lymphocyte ratio, troponin I, etc.) to predict complications in cancer and critically ill patients; 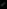 Use of predictive scales based on artificial intelligence to identify the risk of falls, pressure injuries or clinical deterioration; 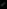 Incorporation of genetic and pharmacogenomic testing into initial assessment, and personalized therapies.
3. Personalized Care Planning	Using the collected data, nurses can develop a patient-centered care plan, taking into account their genomic, environmental, and social profile. This involves: 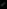 Adjustment of interventions according to patients’ individual response, optimizing treatment effectiveness; 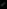 Personalized education and counseling, considering each patient’s profile to improve treatment adherence; 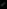 Continuous monitoring and adjustments to the care plan based on patient response.
4. Patient Communication and Education about PN	Nurses play a key role in educating patients and their families about the importance of PN and how it can benefit their treatment. Some strategies include: 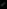 Explaining in an accessible manner the impact of genetic and environmental factors on health; 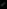 Providing educational materials on genetic testing, biomarkers, and personalized therapies; 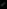 Empowering patients to self-care by encouraging the monitoring of signs and symptoms using digital technologies.
5. Continuous Update and Professional Development	PN evolves rapidly, requiring professionals to seek ongoing training to integrate new approaches into practice. To this end, nurses can: 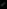 Participate in courses and training on omics sciences and their applicability in healthcare, in addition to the incorporation of artificial intelligence in healthcare. 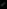 Follow research and guidelines on PN in their area of expertise. 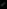 Collaborate with multidisciplinary teams, such as geneticists, pharmacists, and bioinformaticians, to expand knowledge about PN and its applicability in clinical practice.

*PN - precision nursing.*

### Challenges and Perspectives for the Implementation of Precision Nursing

Consolidating PN faces fundamental challenges, the first of which is defining the concept itself and its scope of practice. Globally, there is a growing movement of nurses seeking to define PN, differentiating it from the traditional care model. PN represents the transition from standardized care to a highly personalized approach based on clinical, physiological, environmental, social, and behavioral data^(3)^. This shift emphasizes not only treatment but also health promotion, disease prevention, and early intervention. PN focuses on individualized care, requiring nurses to act with precision and competence when dealing with patients with diverse needs^(3,6,16)^. Therefore, PN is based on the uniqueness of each individual, taking into account their health history, preferences and the best scientific evidence available to offer personalized care throughout the entire life cycle^(4,16)^.

The second major challenge involves preparing and training nurses in omics sciences and disruptive technologies that support personalized care. To achieve this, it is essential to reformulate undergraduate and graduate academic curricula, integrating this knowledge and preparing the workforce to work in PN^(16)^. Continuing education and professional development are essential for nurses to identify areas of application, conduct research, and promote innovation in healthcare^(16)^. Among the areas of greatest development, oncology stands out, in which nurses deal directly with omics sciences for cancer prevention, diagnosis and treatment, using genetic tests, biomarkers, pharmacogenomics, proteomics, and bioinformatics^(3,6,16)^.

Another major challenge concerns the technological incorporation of the “Precision Era”, which raises important ethical questions and impacts on healthcare systems^(16)^. The healthcare paradigm transformation depends on the influence of political, socioeconomic, and cultural factors involving different actors, such as healthcare professionals, researchers, the pharmaceutical industry, managers, patients, and civil society organizations^(16)^. PN advancement has generated intense debate in the field of bioethics, especially regarding access, equity, and cost-effectiveness of new technologies. Therefore, the adoption of these innovations requires a careful assessment of the impacts and feasibility of PN in different healthcare contexts^(16)^.

Finally, a fundamental aspect for PN consolidation is the transfer of knowledge generated by research to clinical practice, facilitating decision-making and promoting personalized care^(16)^. The growth of translational research has enabled significant advances in the understanding of pathophysiological processes, supporting evidence-based clinical practice^(16,17)^. Nursing must actively participate in this movement, conducting research that strengthens evidence-based nursing and contributes to the understanding of biological interactions and their implications for daily care^(3,6,16-24)^. Despite the challenges, translational nursing research can generate significant benefits for patients and their families, positively impacting quality of life and clinical outcomes.

## FINAL CONSIDERATIONS

PN represents an innovative approach with the potential to transform healthcare. However, its implementation requires the development of specialized skills, the incorporation of emerging technologies, and the strengthening of policies that ensure equitable access to innovations. By overcoming these challenges, nursing can establish itself as a leading player in promoting increasingly personalized, efficient, and humanized care.

PN is not a concept far removed from the realities of nursing, but an innovative approach that can be practically and gradually incorporated into daily care. The adoption of technological tools, biomarkers, and individualized plans allows not only anticipating risks and optimizing treatments, but also providing more humanized and effective care. With continuous training and engagement, nurses can lead this transformation, promoting more precise and personalized care for each patient.

## Data Availability

Not applicable.
