# Supplementary material for: Precision Nursing: The Personalized Future of Care and Revolution in Clinical Practice
Source: Rev Bras Enferm. 2026 Mar 30;79:e20250423. doi: 10.1590/0034-7167-2025-0423 (PMC13035284; doi:10.1590/0034-7167-2025-0423)
Supplement: Translation

# Translation: Enfermagem de Precisão: O Futuro Personalizado do Cuidado e a Revolução na Prática Clínica

**DOI:** 10.1590/0034-7167-2025-0423pt

**Authors:** Luís Carlos Lopes-Júnior

**Affiliations:** I Organização Mundial da Saúde, Organização Pan-Americana da Saúde, Departamento de Doenças Não Transmissíveis e Saúde Mental. Washington, D.C., Estados Unidos da América

**Keywords:** Enfermagem de Precisão, Medicina de Precisão, Biomarcadores, Ciência dos Sintomas, Cuidado Personalizado.

## Abstract

**Objetivos:**

discutir os fundamentos, aplicações, desafios e perspectivas da enfermagem de precisão na prática contemporânea, com ênfase na incorporação de tecnologias genômicas, biomarcadores, inteligência artificial e ciência dos sintomas.

**Métodos:**

estudo teórico-reflexivo baseado em evidências científicas recentes.

**Resultados:**

os enfermeiros ocupam posição estratégica para liderar a translação do conhecimento da medicina de precisão para prática clínica, especialmente no manejo de sintomas e na tomada de decisão personalizada. Destacam-se estudos em andamento sobre biomarcadores inflamatórios, distúrbios gastrointestinais, sofrimento sintomático e uso de dispositivos digitais no autocuidado. Persistem desafios éticos, estruturais e formativos, como a reformulação curricular, capacitação profissional e equidade no acesso às inovações.

**Considerações Finais:**

a enfermagem de precisão pode transformar a assistência ao viabilizar intervenções mais eficazes, seguras e centradas na pessoa. É essencial fortalecer a ciência de dados, fomentar a pesquisa translacional em enfermagem e promover sua integração gradual na prática assistencial.

## INTRODUÇÃO

Pesquisadores ao redor do mundo buscam transpor os avanços da bancada laboratorial para a prática clínica e os sistemas de saúde^(1)^. Com base na genômica estrutural, novas técnicas possibilitaram o desenvolvimento da genômica funcional, que se integra às ciências “ômicas”, incluindo proteômica, epigenômica, transcriptômica, farmacogenômica e metabolômica. Essas abordagens investigam as variações no funcionamento do genoma humano ao longo do desenvolvimento e em diferentes contextos ambientais, aprimorando o conhecimento sobre mecanismos moleculares das doenças^(1)^.

A crescente compreensão da biologia molecular impulsionou a transição para a medicina de precisão (MP), um novo paradigma baseado na personalização do tratamento conforme as características genéticas/genômicas individuais ou populacionais^(2)^. A MP reconhece que diferentes grupos apresentam perfis genômicos distintos e propõe intervenções terapêuticas mais eficazes, seguras e custo-efetivas^(1,2)^. Essa abordagem visa oferecer o medicamento certo, na dose exata e no momento adequado, superando as limitações do modelo tradicional de tratamento único para todos (*one-size-fits-all*), que pode ser ineficiente e gerar efeitos adversos^(2)^.

O avanço da MP também influencia a prática de enfermagem, destacando a enfermagem de precisão (EP) como um campo emergente que reforça o papel dos enfermeiros na coordenação, implementação e translação do cuidado personalizado e centrado na pessoa^(3)^. Os enfermeiros, por sua proximidade com os pacientes e conhecimento clínico especializado, estão em uma posição estratégica para liderar essa translação^(3)^. No entanto, desafios significativos ainda precisam ser superados para consolidação da EP na prática clínica, incluindo capacitação profissional, integração de tecnologias genômicas no cotidiano assistencial e equidade no acesso a essas inovações^(3,4)^.

Diante desse cenário, é essencial que a enfermagem se aproprie dos avanços da MP e EP, ampliando sua atuação para proporcionar um cuidado cada vez mais personalizado, preditivo, preventivo e participativo. A incorporação dessas abordagens na prática clínica pode transformar a assistência em saúde, promovendo intervenções mais eficazes e melhorando os desfechos dos pacientes.

## OBJETIVOS

Discutir os fundamentos, aplicações, desafios e perspectivas da EP na prática contemporânea, com ênfase na incorporação de tecnologias genômicas, biomarcadores, inteligência artificial e ciência dos sintomas.

## MÉTODOS

Trata-se de estudo teórico-reflexivo, elaborado a partir da análise crítica do autor, fundamentada em evidências científicas recentes e relevantes sobre EP. A construção do texto seguiu as etapas de: (1) levantamento bibliográfico nas bases MEDLINE/PubMed, Embase, *Web of Science* e Scopus, utilizando descritores controlados e palavras-chave relacionadas a “*precision nursing*”, “*precision medicine*”, “*biomarkers*”, “*symptom science*”, “*personalized care*” , “*personalized nursing care*” e “*omics sciences*”; (2) seleção de artigos, documentos técnicos e diretrizes publicados nos últimos dez anos, priorizando revisões sistemáticas, estudos primários de alta qualidade, posicionamentos de sociedades científicas e relatórios de organizações internacionais; (3) leitura analítica e identificação de categorias temáticas centrais; e (4) integração crítica das evidências com a experiência profissional e acadêmica do autor, visando discutir fundamentos, aplicações, desafios e perspectivas da temática. Essa abordagem permitiu articular a produção científica disponível com reflexões originais, favorecendo uma compreensão ampliada e prospectiva da EP no contexto contemporâneo.

## RESULTADOS

### Avanços Científicos na Enfermagem de Precisão: Aplicações da Pesquisa na Prática Clínica

Enfermeiros no mundo todo vêm expandindo sua atuação na identificação de biomarcadores associados a diferentes condições, como estresse crônico em crianças e adolescentes, fragilidade em idosos, manutenção de acesso vascular, prevenção de lesões por pressão, e comprometimento cognitivo^(5,6)^. Outros profissionais enfermeiros têm explorado inteligência artificial para prever o risco de quedas e lesões por pressão, além de investigar biomarcadores em intervenções não farmacológicas para otimizar desfechos clínicos^(7-9)^. O foco na prevenção, manejo e alívio de sintomas é uma das principais contribuições da ciência da enfermagem para a saúde de precisão voltada à redução de sintomas e melhoria da qualidade de vida^(9,10)^. Incorporar esse conceito nas pesquisas de enfermagem é essencial para aprimorar o desenvolvimento e a aplicação de intervenções ao longo de todo o *continuum* do cuidado^(9,10)^.

Estudo explorou as diferenças raciais observadas nas taxas de recidiva de crianças com leucemia linfoblástica aguda (LLA) para destacar a necessidade de incorporar múltiplos fatores na MP ao estudar desfechos clínicos da doença^(11)^. Embora o papel significativo da genética na LLA seja enfatizado, outros fatores, como determinantes sociais da saúde, também influenciam a precisão dos cuidados^(11)^. Este estudo ressalta a necessidade de pesquisas colaborativas e transdisciplinares para investigar as interações complexas entre múltiplos fatores que contribuem para a MP.

Com base no Modelo de Ciência dos Sintomas do *National Institute of Nursing Research* (NINR)^(12)^, os Centros P20/30 financiados pelo NINR desempenham um papel fundamental na incorporação da ciência dos sintomas nos diferentes domínios da saúde de precisão. Essas iniciativas abrangem abordagens “ômicas”, ciência comportamental e autocuidado, além de considerar os determinantes sociais da saúde para uma compreensão mais abrangente das condições clínicas^(9,12)^.

Vários enfermeiros-cientistas têm desenvolvido pesquisas que utilizam a saúde de precisão para aprimorar o entendimento da carga sintomática, prever e prevenir doenças, bem como otimizar os tratamentos individualizados e a qualidade de vida dos pacientes^(9-13)^. Um exemplo é um estudo conduzido no NINR que investiga os mecanismos envolvidos no sofrimento sintomático em distúrbios digestivos, com ênfase nas relações biocomportamentais entre inflamação e sintomas^(14)^. Esta pesquisa busca identificar os processos biológicos e comportamentais que influenciam a manifestação dos sintomas, destacando o papel do eixo cérebro-intestino-microbiota^(14)^. Os achados indicam que sintomas gastrointestinais crônicos podem estar associados a mecanismos inflamatórios subclínicos^(14)^.

Para viabilizar o pleno desenvolvimento da saúde de precisão, é essencial fortalecer a infraestrutura de ciência de dados tanto em nível institucional quanto nacional. Tradicionalmente, os dados clínicos são obtidos por meio de métodos como questionários padronizados, registros de serviços de saúde, prontuários eletrônicos, relatórios ômicos, exames de imagem e monitoramento fisiológico. No entanto, tecnologias emergentes, como dispositivos móveis de saúde (*mHealth*), sensores não invasivos, como *actigraphy*, e sistemas de monitoramento passivo, têm sido incorporadas para fornecer uma análise mais detalhada das características biológicas, comportamentais e ambientais dos indivíduos^(9,11)^. Um exemplo prático desse avanço pode ser observado na pesquisa sobre diabetes, onde a integração de diferentes fontes de dados permite uma caracterização mais abrangente do fenótipo. Esse processo leva em consideração fatores como estilo de vida, ambiente, perfil genético e biomarcadores. Essas informações são essenciais para personalização das intervenções, tornando o manejo dos sintomas e do autocuidado mais eficaz para indivíduos com maior propensão a responder positivamente às estratégias terapêuticas^(15)^.

### Incorporando a Enfermagem de Precisão na Prática Clínica: Estratégias para o Cotidiano do Enfermeiro

A EP não é uma abordagem distante da prática diária dos enfermeiros; pelo contrário, pode ser integrada de forma simples e progressiva ao cuidado, potencializando a personalização da assistência e otimizando os desfechos em saúde^(3,6,16)^. Para que isso aconteça, os enfermeiros devem adotar estratégias que facilitem a incorporação da EP em diferentes contextos assistenciais. O Quadro 1 ilustra as principais estratégias para implementar na prática clínica a EP.

**Quadro 1:** Estratégias para implementação da enfermagem de precisão, Washington, D.C., Estados Unidos da América, 2025

Estratégias para implementação da enfermagem de precisão na prática clínica do enfermeiro	
1. Coleta e Registro Qualificado de Dados do Paciente	A base da EP está na personalização do cuidado, e isso só é possível com dados detalhados e precisos sobre o paciente. Enfermeiros podem aprimorar a coleta de informações clínicas, considerando: Histórico de saúde detalhado, incluindo predisposição genética, histórico familiar e fatores ambientais; Estilo de vida e hábitos diários, como nutrição, atividade física, padrões de sono e níveis de estresse; Fatores sociais e psicológicos, que podem influenciar a resposta ao tratamento e adesão terapêutica; Uso de tecnologias, como prontuários eletrônicos, dispositivos não invasivos e aplicativos de monitoramento, para registrar e acompanhar dados de forma estruturada.
2. Uso de Ferramentas de Triagem Baseadas em Biomarcadores e Inteligência Artificial	A EP permite que enfermeiros utilizem biomarcadores e recursos tecnológicos para antecipar riscos, prever complicações e personalizar intervenções. Exemplos práticos incluem: Monitoramento de biomarcadores inflamatórios (ex.: proteína-C reativa, razão neutrófilos-linfócitos, razão plaquetalinfócitos, troponina I, etc.,) para prever complicações em pacientes oncológicos e críticos; Uso de escalas preditivas baseadas em inteligência artificial para identificar risco de quedas, lesões por pressão ou deterioração clínica; Incorporação de testes genéticos e farmacogenômicos na avaliação inicial, e terapias personalizadas.
3. Planejamento de Cuidados Personalizados	A partir dos dados coletados, o enfermeiro pode elaborar um plano de cuidados centrado no paciente, levando em conta seu perfil genômico, ambiental e social. Isso envolve: Ajuste de intervenções conforme a resposta individual do paciente, otimizando a eficácia do tratamento; Educação e aconselhamento personalizado, considerando o perfil de cada paciente para melhorar a adesão ao tratamento; Monitoramento contínuo e ajustes no plano de cuidados com base na resposta do paciente.
4. Comunicação e Educação do Paciente sobre a EP	Os enfermeiros têm um papel fundamental na educação dos pacientes e suas famílias sobre a importância da EP e como ela pode beneficiar seu tratamento. Algumas estratégias incluem: Explicar de forma acessível o impacto de fatores genéticos e ambientais na saúde; Oferecer materiais educativos sobre testes genéticos, biomarcadores e terapias personalizadas; Capacitar os pacientes para o autocuidado, incentivando o monitoramento de sinais e sintomas com uso de tecnologias digitais.
5. Atualização Contínua e Desenvolvimento Profissional	A EP evolui rapidamente, exigindo que os profissionais busquem capacitação contínua para integrar as novas abordagens à prática. Para isso, o enfermeiro pode: Participar de cursos e treinamentos sobre as ciências ômicas e sua aplicabilidade no cuidado, além da incorporação da inteligência artificial na saúde; Acompanhar pesquisas e diretrizes sobre EP em sua área de atuação; Colaborar com equipes multidisciplinares, como geneticistas, farmacêuticos e bioinformatas, para ampliar o conhecimento sobre EP e sua aplicabilidade na prática clínica.

*EP - enfermagem de precisão.*

### Desafios e Perspectivas para a Implementação da Enfermagem de Precisão

A consolidação da EP enfrenta desafios fundamentais, sendo o primeiro deles a própria definição do conceito e seu escopo de prática. Globalmente, há um movimento crescente de enfermeiros que buscam delimitar a EP, diferenciando-a do modelo tradicional de cuidados. A EP representa a transição de um atendimento padronizado para uma abordagem altamente personalizada baseada em dados clínicos, fisiológicos, ambientais, sociais e comportamentais^(3)^. Essa mudança enfatiza não apenas o tratamento, mas também a promoção da saúde, prevenção de doenças e intervenções precoces. A EP se centra no atendimento individualizado, exigindo que enfermeiros atuem com precisão e competência ao lidar com pacientes que apresentam necessidades diversas^(3,6,16)^. Dessa forma, a EP fundamenta-se na singularidade de cada indivíduo, levando em consideração seu histórico de saúde, preferências e as melhores evidências científicas disponíveis para oferecer um cuidado personalizado ao longo de todo o ciclo de vida^(4,16)^.

O segundo grande desafio envolve a preparação e capacitação dos enfermeiros nas ciências ômicas e nas tecnologias disruptivas que sustentam o cuidado personalizado. Para isso, é essencial reformular os currículos acadêmicos da graduação e pós-graduação, integrando esses conhecimentos e preparando a força de trabalho para atuar na EP^(16)^. A formação continuada e aprimoramento profissional são indispensáveis para que enfermeiros possam identificar áreas de aplicação, conduzir pesquisas e promover inovação em saúde^(16)^. Entre as áreas de maior desenvolvimento, destaca-se a oncologia, na qual enfermeiros lidam diretamente com as ciências ômicas para prevenção, diagnóstico e tratamento do câncer, utilizando testes genéticos, biomarcadores, farmacogenômica, proteômica e bioinformática^(3,6,16)^.

Outro grande desafio refere-se à incorporação tecnológica da “Era da Precisão”, que levanta importantes questões éticas e impactos para os sistemas de saúde^(16)^. A transformação do paradigma assistencial depende da influência de fatores políticos, socioeconômicos e culturais que envolvem diferentes atores, como profissionais de saúde, pesquisadores, indústria farmacêutica, gestores, pacientes e organizações da sociedade civil^(16)^. O avanço da EP tem gerado intensos debates no campo da bioética, especialmente sobre acesso, equidade e custo-benefício das novas tecnologias. Portanto, a adoção dessas inovações exige uma avaliação criteriosa dos impactos e da viabilidade da EP nos diferentes contextos de atenção à saúde^(16)^.

Por fim, um aspecto fundamental para a consolidação da EP é a transferência do conhecimento gerado por pesquisas para prática clínica, facilitando a tomada de decisão e promovendo o atendimento personalizado^(16)^. O crescimento da pesquisa translacional tem permitido avanços significativos no entendimento de processos fisiopatológicos, apoiando a prática clínica baseada em evidências^(16,17)^. A enfermagem deve se inserir ativamente nesse movimento, conduzindo investigações que fortaleçam a enfermagem baseada em evidências e contribuam para a compreensão das interações biológicas e suas implicações no cuidado diário^(3,6,16-24)^. Apesar dos desafios, a pesquisa translacional em enfermagem pode gerar benefícios expressivos para pacientes e suas famílias, impactando positivamente a qualidade de vida e os desfechos clínicos.

## CONSIDERAÇÕES FINAIS

A EP representa uma abordagem inovadora, com potencial para transformar o cuidado em saúde. Contudo, sua implementação requer o desenvolvimento de competências especializadas, a incorporação de tecnologias emergentes e o fortalecimento de políticas que garantam equidade no acesso às inovações. Ao superar esses desafios, a enfermagem poderá se consolidar como protagonista na promoção de um cuidado cada vez mais personalizado, eficiente e humanizado.

A EP não é um conceito distante da realidade dos enfermeiros, mas uma abordagem inovadora que pode ser incorporada de forma prática e gradual ao cuidado diário. A adoção de ferramentas tecnológicas, biomarcadores e planos individualizados permite não apenas antecipar riscos e otimizar tratamentos, mas também proporcionar um cuidado mais humanizado e eficaz. Com capacitação contínua e engajamento, os enfermeiros podem liderar essa transformação promovendo uma assistência mais precisa e personalizada para cada paciente.

## Data Availability

Não se aplica.
